# The Anatomical Relationships of the Tongue with the Body System

**DOI:** 10.7759/cureus.3695

**Published:** 2018-12-05

**Authors:** Bruno Bordoni, Bruno Morabito, Roberto Mitrano, Marta Simonelli, Anastasia Toccafondi

**Affiliations:** 1 Cardiology, Foundation Don Carlo Gnocchi / Institute of Hospitalization and Care, Milan, ITA; 2 Osteopathy, School of Osteopathic Centre for Research and Studies, Milan, ITA; 3 Cardiology, Foundation Don Carlo Gnocchi/institute of Hospitalization and Care, Milan, ITA; 4 Osteopathy, School of French-Italian Osteopathy, Pisa, ITA

**Keywords:** tongue, diaphragm, speech therapy, osteopathic, physiotherapy, fascia

## Abstract

The tongue plays a fundamental role in several body functions such as swallowing, breathing, speaking, and chewing. Its action is not confined to the oral cavity, but it affects lower limb muscle strength and posture. The tongue is an organ that has an autocrine/paracrine mechanism of action to synthesize different substances to interact with the whole body; according to a line of thought, it is also an extension of the enteric system. The aim of this study was to review the functions of the tongue and its anatomical association with the body system. According to the authors' knowledge, this is the first scientific article focusing on the tongue in a systemic context. In a clinical evaluation, connections with the tongue should be considered to optimize the clinical examination of the tongue and therefore enhance rehabilitation programs and therapeutic results.

## Introduction and background

The tongue is an area of interest for different professional profiles. Alterations in the tongue muscle properties are associated with different pathological conditions. Systemic disorders may involve secondarily the tongue, or local tongue alterations can cause systemic diseases: scleroderma, local tongue dysfunction, tumors, old age, obstructive sleep apnea syndrome (OSAS), and emotional disorders.

Scleroderma

Scleroderma is a chronic connective tissue disease generally classified as one of the autoimmune rheumatic diseases, presenting with vascular anomalies and antibody production; scleroderma most frequently affects women compared to men (4:1); its pathogenesis remains not entirely understood [1]. Scleroderma is classified as two separate but related entities, a localized form and a systemic form: systemic sclerosis and morphea (localized scleroderma) [2]. The tongue is involved in scleroderma, with changes in its form and function, i.e., the tongue becomes rigid with less ability to perform its functions, which can cause problems that may affect the temporomandibular joint (TMJ), dysphagia, dysgeusia, and logopedic problems [1].

Local tongue dysfunction

In the absence of pathological conditions, altered tongue function leads to TMJ disorders and pain [3]. The most likely explanation for this phenomenon could be a dysfunction of the genioglossus muscle activity during the jaw-closing phases, suggesting a peripheral mechanism (jaw-tongue reflexes or afferent fibers from the extrinsic muscles of the tongue), or a central organization (a central cortical generator that controls the mastication process), or both neural mechanisms [4].

Tumors

The tongue is a common site for tumors involving the oral cavity and the cervical tract. One of the most frequent intraoral areas affected by carcinomas is the tongue, occurring after the age of 40 on average, with men affected more than women (10%); however, the causes of tongue cancer still remain unclear [5]. Chemotherapy treatments will lead to dysphagia, loss of tongue strength and tone, and dysgeusia [6].

Old age

In patients requiring long-term management, from the hospital to home setting (Parkinson's disease, stroke, and dementia), especially in the elderly population, there is a direct association between the lean mass loss (muscles) and the lingual discoordination; the decline in tongue motor skills has the same characteristics of the voluntary skeletal muscle degeneration [7-8]. Usually, an age-related loss of the tongue muscle mass and strength exists in healthy subjects, with an increased adiposity in the posterior area; after the age of 55, an age-dependent motor unit remodeling occurs, with a tongue functional impairment [9-10].

Obstructive sleep apnea syndrome

OSAS is a systemic disease that involves the tongue; 2% of women and 4% of men present this disorder, especially in the middle-aged population. OSAS is the most common type of sleep apnea or hypopnea and is caused by complete or partial obstructions of the upper airway. It is characterized by repetitive episodes of shallow or paused breathing during sleep and is usually associated with a reduction in blood oxygen saturation and hypercapnia [11-12]. The most important cause of this syndrome is the dysfunction of the lingual muscles, with changes in shape and altered electrical activation [12].

Emotional disorders

The tongue position changes with emotions and state of mind, thus becoming an instrument for psychological observation; usually, an anterior tongue placement occurs with a feeling of fear [13]. The multidisciplinary collaboration between the different professional profiles is fundamental to obtaining an appropriate therapeutic effect.

## Review

Anatomic connections of the tongue

The cell origin of the tongue is hybrid. The connective tissues and the vascular system are derived from the cranial neural crest cell-derived mesenchyme; the tongue muscle cells originate from the somite mesoderm, while the muscles of mastication are derived from the unsegmented somitomeres. Lingual musculature stems from muscle cells that immigrate from the occipital somites into the tongue [7]. A close embryological and functional association exists among the tongue, the occipital area, and the hyoid bone that originates from the second branchial arch [7]. Anatomically, the tongue maintains several relations with the hyoid bone, and therefore, with the hyoid musculature (the suprahyoid and the infrahyoid muscles) [7]. The hyoglossus membrane and the lingual septum bind the tongue to the hyoid muscles [8]. The suprahyoid muscular action helped in maintaining the posture and the equilibrium of the head. Electromyography showed electrical activity in the omohyoid muscle and in the anterior belly of the digastric muscle during different movements of the tongue; these muscles intervene to allow a proper association between the tongue and the head (neck), during flexion, extension, and rotation of the neck and the cervical tract [8]. The suprahyoid and the infrahyoid muscles act together in the jaw and tongue movements, during the first phase of swallowing and phonation [8]. These two muscles and the tongue act simultaneously in all the tongue movements (except retraction); the lingual musculature and the suprahyoid and the infrahyoid muscles influence each other [8]. Chewing involves an anterior-posterior movement of the tongue and of the hyoid bone on the horizontal plane, whereas the hyoid bone has almost no role in speaking [7]. During respiration, the hyoid bone moves in a craniocaudal direction, due to the action of the extrinsic muscles of the tongue, causing the pharyngeal space to dilate [7]. Generally, the anterior part of the tongue is considered important for non-respiratory activities, while the posterior part is important for respiration [9]. It should be emphasized that all the tongue muscles, extrinsic and intrinsic muscle groups, always work synergistically and not separately [9]. The tonus of these muscles must be well-balanced; otherwise, dysfunctions can occur, resulting in an alteration in the position of the hyoid bone and the functionality of the tongue [3,9-10]. The suprahyoid region extends from the base of the skull to the hyoid bone and includes the pharyngeal, parapharyngeal, parotid, carotid, masticator, retropharyngeal, and perivertebral spaces, as well as the oral cavity [11]. The tongue is a part of the anatomical structures that cover the occipitocervical area, the anterior area of the neck, and the muscles of mastication, including the temporomandibular joint, as well as the three layers of the cervical fascia (superficial, middle, and deep) [11]. The suprahyoid region and the tongue have some muscles in common: the masseter, the buccinator, the temporalis, the pterygoid, and the mylohyoid [11]. The floor of the oral cavity is formed by the mylohyoid muscle, a quadrilateral sheet consisting of two bellies, whose lower fasciae are in contact with the anterior fascia of the digastric muscle and superiorly with the geniohyoid and hyoglossus muscles; with a connective raphe and a sagittal fibrous lamina, the mylohyoid muscle contacts the hyoid bone. The infrahyoid region is located below the hyoid bone and continues into the suprahyoid region. The deep cervical fascia extends from the hyoid bone to the upper mediastinum, continuing along its path toward the visceral, anterior cervical, posterior cervical, carotid, retropharyngeal, and perivertebral spaces [12]. For fascial continuity, the tongue is connected to the strap muscles and the sternocleidomastoid muscle and to the musculature acting on the thoracic outlet [12-13]. The tongue can change its shape and produce different actions working as a hydrostat; all the muscular components, in direct or indirect contact with the tongue, respond with proper contractile tonus to allow the tongue to work properly, thanks to the complex organization of the central and peripheral nervous system [14-15].

Neurological relationships

Studies demonstrated the highly organized and extensive representation of the tongue at multiple levels in the brain (cortex, mesencephalon, medulla oblongata, and limbic system), with the highest specificity and integration reached at the cortical level, with a clear somatotopic organization [16]. Neuroplastic changes in the cortex demonstrate how the brain control of the tongue activity can be modified by environmental stimuli: an improvement in functions occurs in case of physiological stimuli; instead, functional disorders occur with pathological stimuli [17] These stimuli are given by the position of the tongue inside the mouth, as described later. Afferent nerve fibers of the peripheral nervous system send information to the central nervous system, and the information is able to oscillate between one hemisphere and another, to ensure the optimal tongue movement efficiency [18]. The tongue position influences the whole body. If the tongue is positioned against the palate, the parasympathetic system will reduce its systemic activity (for example, heartbeats and respiratory rhythm increase), but if it is positioned against the soft palate, the sympathetic system will reduce its activity [17]. The tongue position and its voluntary (or non-voluntary) strength might vary with the lung volume. Changes in the tracheal traction at different lung volumes may alter the mechanics of the tongue muscles and their ability to produce protrusion force, and these changes in the lung volume alter tension transferred through the trachea to the hyoid arch. Pulmonary stretch activates the pleural receptors that work by inhibiting the hypoglossal/phrenic motoneurons [18-19]. It is assumed that when a chronic pathology takes over, restricting lungs expansion and decreasing lungs volume, the hypoglossal/phrenic motoneurons are less inhibited, resulting in a reduced tongue control [20]. Tongue movements, generally postero-lateral, activate the anterior cingulate cortex (ACC), which plays an important role in the sensory, motor, cognitive, and emotional information and pain processing; some studies using magnetoencephalography showed the ACC to often be concerned with visceral sensation [21]. Lingual behavior has a link with the amygdala, especially for taste learning, forming motor patterns during mastication [22]. The amygdala has multiple functions regulating mood and emotions [23]. According to some authors, the tongue represents the gateway to the enteric system, due to the presence of specialized ganglia on the anterior and posterior area of the tongue; it would act as a chemical "spy", alerting the enteric system through the chemical analysis of food during mastication [24]. In this way, the digestive system would already be prepared, before the food arrives in the stomach and the intestine; the tongue would modulate the digestive functions [24].

Systemic relationships

The tongue has autocrine and paracrine functions, modulating the function of the tongue muscles and the surrounding environment. Type II receptor taste bud cells secrete adenosine triphosphate (ATP) during taste stimulation. In turn, ATP activates the type III cells to release serotonin, norepinephrine, and gamma-aminobutyric acid (GABA) [25]. They also synthesize acetylcholine that also stimulates ATP secretion by releasing calcium into the Type II receptors; through these autocrine strategies, the tongue modulates gustatory signals that are transmitted to the central nervous system [25]. Perhaps, ATP is secreted via the paracrine mechanism to stimulate the systemic pathways for adenosine production; the tongue needs adenosine to recreate ATP [26]. In the animal model, the tongue has a sympathetic innervation from the superior cervical ganglion, whose fibers provide sympathetic innervation to the whole tongue [27]. Most likely, the human tongue also has sympathetic innervations, but less is known in this regard [28]. The tongue influences the neuromotor control of the lower limb. A pilot study showed a significant improvement in the isokinetic knee performance with the tongue positioned up to the palatine spot, increasing the performance of thigh muscles (through the use of an isokinetic machine), for about +30% with respect to the resting position (during both endurance and high-force muscular exercise) [29]. The palatine spot has a high density of trigeminal nerve endings and exteroceptors [29]. Electrical tongue stimulation improves balance, gait, and posture in subjects with postural impairments [30]. A hypothesis to explain these behavioral changes is based on the neuroanatomy of the trigeminal system (V) and facial nerve (VII). The brainstem projections of these nerves are the trigeminal and solitary nuclei, located immediately adjacent to the vestibular nuclei near the dorsal aspect of the pontomedullary junction and extending superiorly through the pons; electrical stimulation of these cranial nerves may induce modulating activity in the vestibular system [30]. The body position induces changes in the tongue's myoelectric activity; tonic tongue muscle activity and movements related to spontaneous respiration increase significantly in the supine position with respect to the upright position, thus maintaining an adequate upper airway patency [31]. A study shows that the mean center of gravity (COG) velocity decreased significantly while the tongue was positioned against the upper incisors, suggesting that this tongue position can enhance the postural stability during upright standing on an unstable surface and in the absence of vision in healthy young adults [32]. Another study investigated the improvement of head postural control in the absence of visual sensory cues, through an electrical stimulation of the tongue with a biofeedback [33]. The tongue has control on the posture, thanks to its greater tactile sensitivity than the finger; besides, compared with other body parts, the tongue is represented by the large primary motor and sensory cortical areas [34]. The tongue position would interact with the body posture in the context of postural dysfunction. We know that the tongue affects the occlusal class and that there exists a relationship between the occlusal class and the pathological postures; during child growth or fetal development, the tongue could cause postural modifications, altering systemic tensions by fascial or trigeminal connections [35-37]. Tongue representation in the primary somatosensory cortex is activated, during observation of video sequences of bimanual hand movements associated with nouns; the lingual complex interacts with what we observe, influencing the same speech, through a neurological relationship not yet elucidated, suggesting a greater complexity of the tongue functions [38]. The human tongue innervation involves the lingual nerve that is a branch of the mandibular division of the trigeminal nerve and the hypoglossal nerve (CN XII) [39]. The hypoglossal nerve through the ansa cervicalis is in contact with the first three or four cervical nerves and receives presynaptic impulses from the phrenic nerve; it is linked to the trigeminal system by afferent fibers [40]. The tongue becomes a crossroad of efferent and afferent information, and its dysfunction will negatively affect all the systems. For example, a chronic decline in the heart function (chronic heart failure: CHF) alters the morphology and function of the diaphragm with a concomitant functional alteration in the tongue, causing OSAS [41]. An altered function of the tongue negatively affects the TMJ; consequently, a dysfunctional TMJ will negatively affect the trigeminocardiac reflexes, altering values such as heart rate (bradycardia) and blood pressure (hypotension) [42]. The morphology and the non-physiological position of the tongue negatively affect renal function, smell and hearing, and the cognitive area [43-45]. The causes are probably related to an incorrect breathing at night connected to tissue deoxygenation.

## Conclusions

The tongue influences and interacts with the body system. Its dysfunction leads to different local and systemic pathologies. During the assessment of the tongue, other associations that may influence its physiological behavior, such as the lower limb, the TMJ, the neck, the respiratory, and the pelvic diaphragm, and the muscles of the thoracic outlet, should not be neglected. Considering its anatomical and physiological connections, during manual evaluation, will help the operators increase the importance of the tongue assessment, the rehabilitation organization, and consequently, the therapeutic results.
